# Betulinic acid impairs metastasis and reduces immunosuppressive cells in breast cancer models

**DOI:** 10.18632/oncotarget.23376

**Published:** 2017-12-17

**Authors:** An-Qi Zeng, Yan Yu, Yu-Qin Yao, Fang-Fang Yang, Mengya Liao, Lin-Jiang Song, Ya-Li Li, Yang Yu, Yu-Jue Li, Yuan-Le Deng, Shu-Ping Yang, Chen-Juan Zeng, Ping Liu, Yong-Mei Xie, Jin-Liang Yang, Yi-Wen Zhang, Ting-Hong Ye, Yu-Quan Wei

**Affiliations:** ^1^ Laboratory of Liver Surgery, State Key Laboratory of Biotherapy, West China Hospital, Sichuan University and Collaborative Innovation Center for Biotherapy, Chengdu 610041, China; ^2^ Sichuan Nursing Vocational College, Chengdu 610100, China; ^3^ Department of Peritoneal Cancer Surgery, Beijing Shijitan Hospital Affiliated to the Capital Medical University, Beijing 100038, China; ^4^ Sichuan Scientist Biotechnology Co., Ltd, Chengdu 610041, China; ^5^ Department of Gynecology and Obstetrics, Key Laboratory of Birth Defects and Related Diseases of Women and Children of the Ministry of Education, West China Second Hospital, Sichuan University, Chengdu 610041, China

**Keywords:** betulinic acid, breast cancer, migration and invasion, pulmonary metastases, MDSCs

## Abstract

Breast cancer is the most common female cancer with considerable metastatic potential, explaining the need for new candidates that inhibit tumor metastasis. In our study, betulinic acid (BA), a kind of pentacyclic triterpenoid compound derived from birch trees, was evaluated for its anti-metastasis activity *in vitro* and *in vivo*. BA decreased the viability of three breast cancer cell lines and markedly impaired cell migration and invasion. In addition, BA could inhibit the activation of stat3 and FAK which resulted in a reduction of matrix metalloproteinases (MMPs), and increase of the MMPs inhibitor (TIMP-2) expression. Moreover, in our animal experiment, intraperitoneal administration of 10 mg/kg/day BA suppressed 4T1 tumor growth and blocked formation of pulmonary metastases without obvious side effects. Furthermore, histological and immunohistochemical analyses showed a decrease in MMP-9 positive cells, MMP-2 positive cells and Ki-67 positive cells and an increase in cleaved caspase-3 positive cells upon BA administration. Notably, BA reduced the number of myeloid-derived suppressor cells (MDSCs) in the lungs and tumors. Interestingly, in our caudal vein model, BA also obviously suppressed 4T1 tumor pulmonary metastases. These findings suggested that BA might be a potential agent for inhibiting the growth and metastasis of breast cancer.

## INTRODUCTION

According to statistics, the three most commonly diagnosed types of cancer in women were breast, lung and colorectum in the USA in 2015. In particular, breast cancer, alone is expected to account for approximately 29% of all yearly diagnosed cancer cases in women [1]. Moreover, the occurrence of breast cancer-like most cancer is step up in developing countries such as Brazil and China [2]. Although progresses have been made in breast cancer, the huge threat of this cancer for women is still serious, and particularly the patients who suffer from the ‘triple-negative’ breast cancer (TNBC), which is negative for the expressions of progesterone (PR), estrogen receptors (ER) and HER2 [3–5]. The terminal TNBC have an aggressive clinical progress with a bad prognosis compared with non-TNBC [6]. Besides, highly malignant and metastatic of breast cancer is a principle cause of female mortality [7]. Unfortunately, there is no effective therapy to control the reappearance and metastasis of breast cancer. Thus, finding potent and effective candidates for metastatic breast cancer with potential anti-tumor activity and low toxicity is necessary.

Natural products have been used to treat human diseases for thousands of years and have precious values in drug discovery and development [8, 9]. Most of anti-cancer and anti-infectious agents are derived from natural products [9]. Furthermore, in recent decades, the rapid development of more effective drugs with fewer, less-severe side effects is a common goal shared by scientists and clinicians [10]. Therefore, the identification of the naturally occurring phytocompounds from medicinal plants and natural products to combat diseases has become crucial due to its low side effects [11]. Betulinic acid (BA)(Supplementary Figure 1) (3β, hydroxyl-lup-20(29)-en-28-oic acid) is a natural pentacyclic triterpene with a lupine structure, which is found in plant sources including various bark extractant, acuminatissima leaves, and wild jujube seeds [8, 12, 13]. Studies have demonstrated that BA has a variety of beneficial biological activities, including anti-cancer, anti-viral, anti-bacterial, anti-malarial and anti-inflammatory activity [14–16]. Moreover, BA could suppress tumorigenesis in various types of cancer, including breast, lung, colon, pancreatic, and prostate cancer [17–21].

As to breast cancer, it is reported that BA can inhibit viability of breast cancer cells and angiogenesis, and induce mitochondrial pathway of cell apoptosis [17, 22]. The anti-tumor mechanism of BA in breast cancer cells have been reported, at least including topoisomerase [23], transcription factor nuclear factor kappa B (NF-kB) [24], vascular endothelial growthfactor (VEGF) and its receptor (VEGFR) [25], and multidrug resistance proteins [26]. However, the effects of BA on suppressing pulmonary metastasis of breast cancer and its related molecular mechanism have not yet been reported. Considering the effects of BA on breast cancer, we hypothesized that BA might inhibit the metastasis of breast cancer. Therefore, the anti-proliferation and anti-metastasis efficacy of BA *in vitro* and *in vivo* were assessed in the current study. Furthermore, the anti-metastasis mechanism of BA were also explored.

## RESULTS

### BA inhibits breast cancer cells proliferation

**Figure 1:**
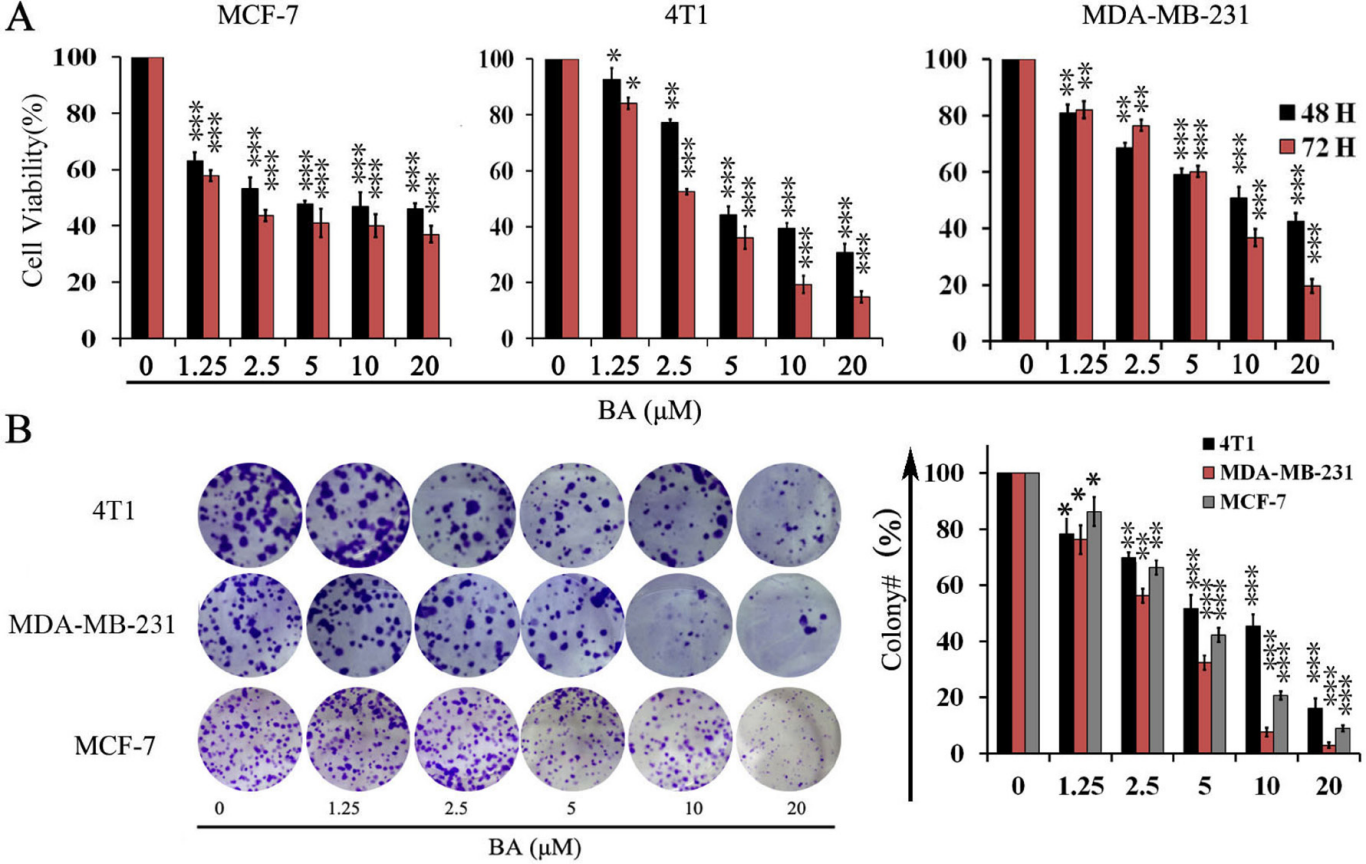
The effects of BA in breast cancer cells viability (**A**) Proliferation of MCF-7, 4T1 and MDA-MB-231 cells treated with various concentrations (0–20 μM) of BA for 48 h and 72 h. Cell viability was evaluated by MTT assay. Data represent mean ± SD at least from 3 independent experiments. (**B**) The effects of BA (0–20 μM) on colony formation in 4T1 and MDA-MB-231 cell lines for 12 days, the statistic results of colony formation assays presented surviving colonies. Data are expressed as mean ± SD at least from 3 independent experiments (^*^P < 0.05; ^**^P < 0.01; ^***^P < 0.001).

To evaluate whether BA has direct effects on breast cancer cells, we treated three established breast cancer cell lines with BA and detecting cell viability by 3-(4, 5)-dimethylthiahiazo(-z-y1)-3,5-di-phenytetrazoliumromide(MTT). Treatment of MCF-7, 4T1 and MDA-MB-231 cells with various concentration of BA for 48 and 72 h respectively, resulted in a concentration-dependent decrease in the cell viability (Figure 1A). These data suggested that BA could inhibit breast cancer cells viability in a concentration and time-dependent manner. To further examine whether BA could inhibit proliferation of breast cancer cells, we conducted clonogenic assay to visually assess the anti-proliferation activity of BA. As shown in Figure 1B, clonogenic assay definitely showed that clone formation of 4T1, MCF-7 and MDA-MB-231 cells was significantly reduced in a concentration-dependent manner after exposure to BA. Meanwhile, the size of the colonies treated with BA was significantly smaller than the control group. These results were consistent with the MTT data. Taken together, those resultsconfirmed that BA had a strong cytostatic and cytotoxic effect on breast cancer cells.

### BA inhibits breast cancer cell migration and invasion

**Figure 2:**
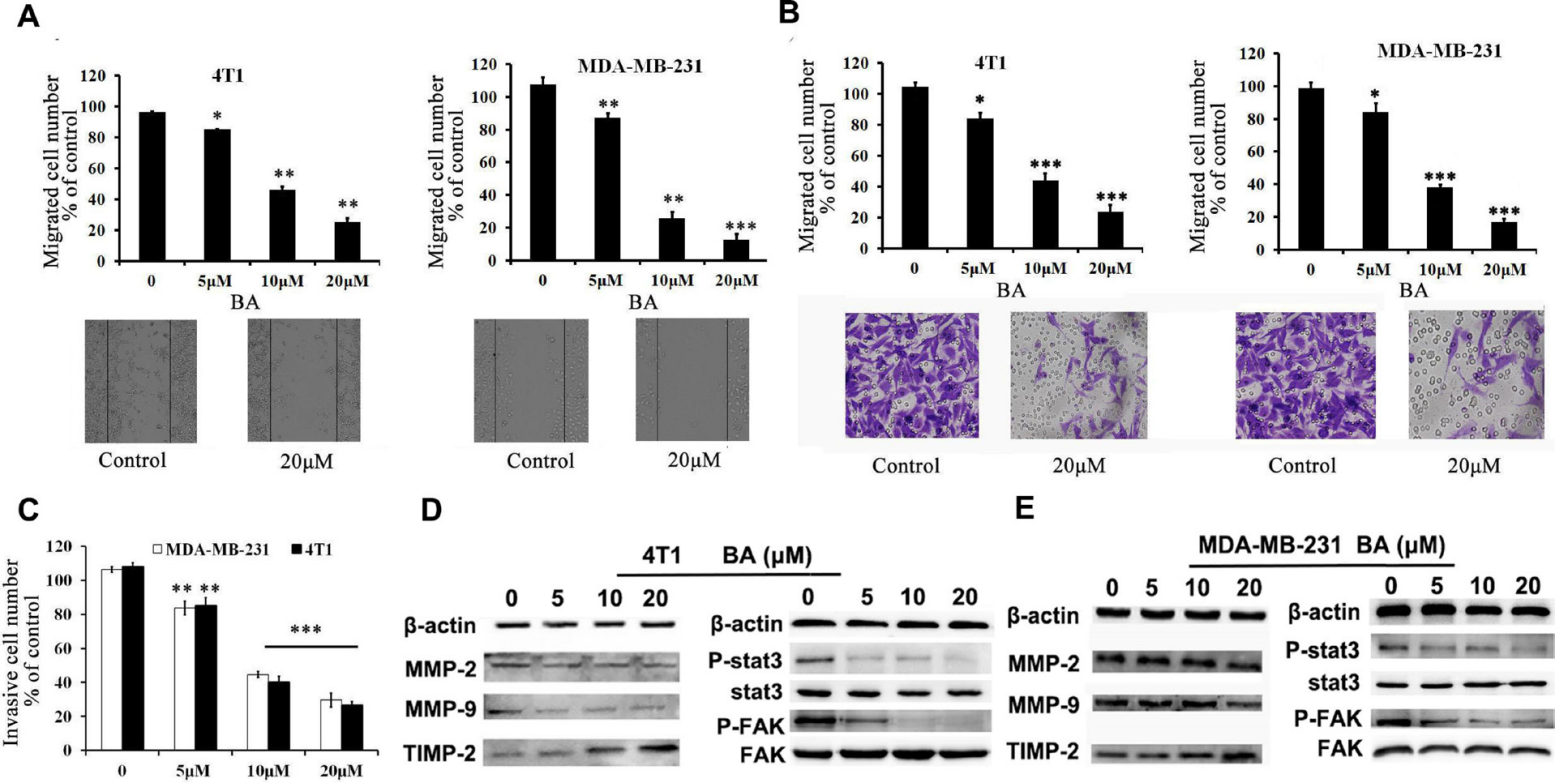
BA inhibits breast cancer cells 4T1 and MDA-MB-231 migration and invasion (**A**) Tumor cells were seeded in six-well plates. We make a ’wound’ after the cells grew ∼90% confluence. After incubation for 48 h, the groups were graphed. The black lines indicate the section occupied by the initial scraping, and migrated cells were quantified. (**B**) Tumor cells were seeded in the roof chamber of transwell with serum-free medium and treated with vehicle or different concentrations of BA. After 48 h, migrated cells were fixed, stained and graphed (20×) and quantified. (**C**) BA inhibits 4T1 and MDA-MB-231 invasion. Tumor cells were treated with different concentrations of BA and invaded through Matrigel. Invaded cell number was counted (^*^P < 0.05; ^**^P < 0.01; ^***^P < 0.001). (**D**, **E**) 4T1 and MDA-MB-231 cells were treated with different concentrations of BA. After 48 h, cells were harvested, and western blot assay was performed to detect the expression of MMP-2, MMP-9, TIMP-2, Stat3, P-Stat3, FAK, P-FAK. β-actin served as loading control.

Migration and invasion of tumor cells are regarded as a crucial step for initial breast cancer metastasis [27, 28]. Therefore, it is necessary to investigate whether BA could inhibit breast cancer cell migration and invasion. To test the effects of BA on migration, we performed wound-healing and transwell migration assays using 4T1 and MDA-MB-231 cell lines. As shown in Figure 2A, BA inhibited migration of both 4T1 and MDA-MB-231 cells in dose-dependent manners. Similar results were obtained in transwell migration assays (Figure 2B and Supplementary Figure 2). Furthermore, we made a Matrigel invasion assay. Figure 2C showed that both 4T1 and MDA-MB-231 cells exhibit significantly decreased invasion in the presence of BA than control groups. Matrix metalloproteinases (MMPs) have been identified as the major molecules in cancer metastasis [27]. Stat3 and Src/FAK/Rac1 signal are known as an vital role in controlling cell migration and invasion by regulating the expression level of genes included MMP2, 9 and it is established that the level of MMPs is positively related to cancer cell metastasis. [28, 29] Therefore, we also investigated whether MMP-2, MMP-9 and TIMP-2 are considered to be related with cell migration and invasion by BA. As Figure 2D, 2E and Supplementary Figure 3 indicated, BA treatment decreased the expression of MMP-2 and MMP-9 while increased the expression of TIMP-2 in 4T1 and MDA-MB-231 cells. Moreover, The results of western blot indicated that the treatment with BA significantly inhibited the levels of p-Stat3 and P-FAK in 4T1 and MDA-MB-231 cells(Figures 2D, 2E and Supplementary Figure 3). Altogether, all of these results indicated that BA possessed a strong ability on breast cancer cell migration and invasion.

### Anti-metastasis efficacy of BA in subcutaneous 4T1 model

**Figure 3:**
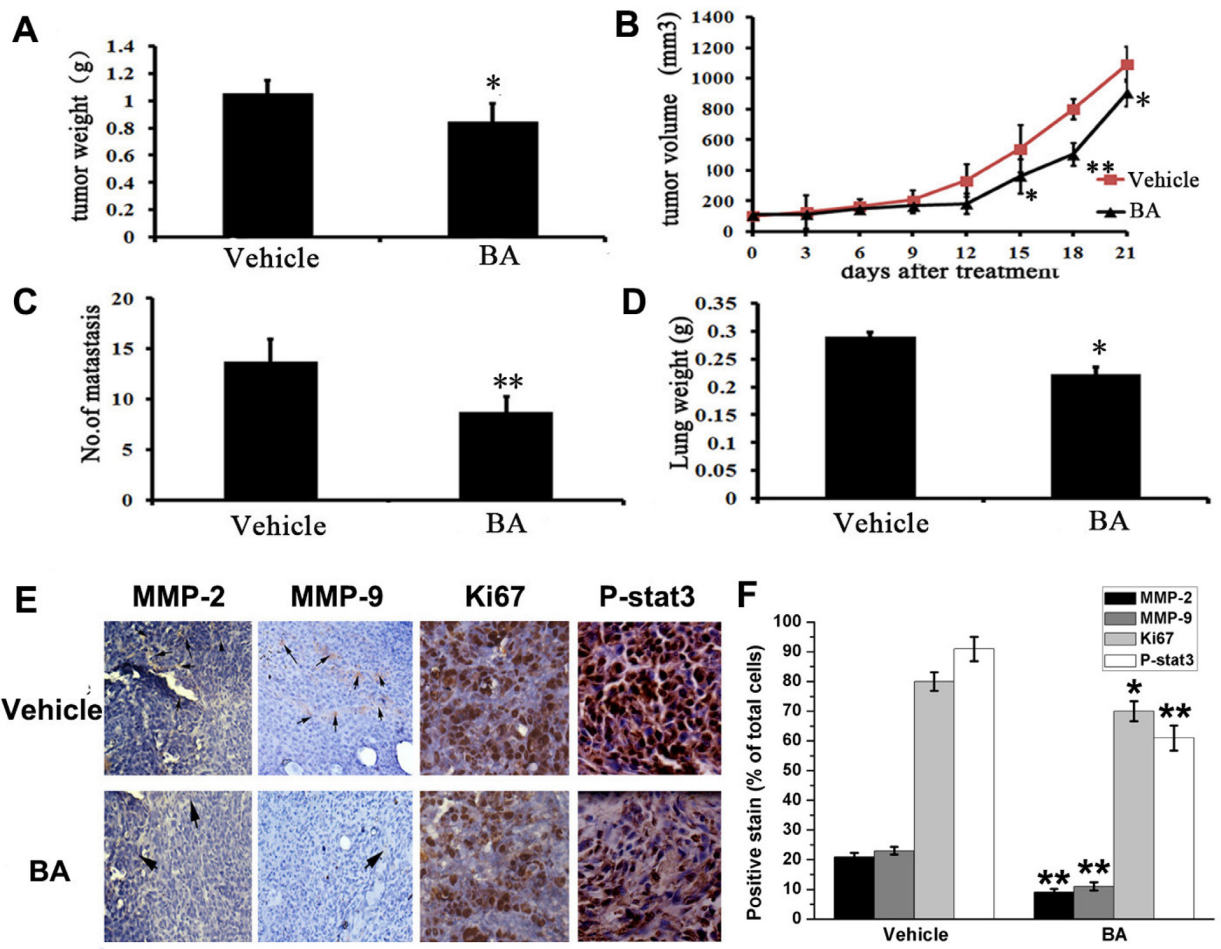
Anti-tumor and anti-metastasis effects of BA *in vivo* (**A**) Represented weight of tumor from mice of different groups (vehicle and BA (10 mg/kg/day)), respectively. Data were mean ± SD (*n =* 6; ^*^P < 0.05). (**B**) Tumor volume were measured and calculated every three days and presented as mean ± SD (*n =* 6; ^*^P < 0.05, ^**^P < 0.01). (**C**) The number of metastasis from lungs. Data were mean ± SD (*n =* 6; ^*^P < 0.05). (**D**) The lungs weight of different groups. Results were mean ± SD (*n =* 6; ^**^P < 0.01). (**E**) Immunohistochemistry was performed to measure the expression of MMP-2, MMP-9, Ki67 and P-Stat3 in tumor tissues isolated from vehicle and BA-treated (10 mg/kg/day) mice. The treatment with BA markedly reduced MMP-2, MMP-9, Ki67 and P-Stat3-positive cells versus vehicle group (20×).

The remarkable inhibitory effects of BA on 4T1 cells metastasis *in vitro* implied that it might also efficiently inhibit tumor metastasis *in vivo*. To verify this hypothesis, we investigated the anti-metastasis activity of BA *in vivo*. Firstly, we used a subcutaneous model using 4T1 cells. Then the tumor-bearing mice were treated daily at the dose of 10 mg/kg for 21 days. There was not different body weight of mice between BA-treated and vehicle-treated groups (Supplementary Figure 4D). However, there was a reduction in tumor growth and tumor weight in the BA-treated groups (Figure 3A, 3B, Supplementary Figure 4A and Supplementary Figure 4C). Moreover, the number of lung metastatic nodules and lungs weight were both decreased after treating by BA (Supplementary Figure 4B, Figure 3C and 3D). In addition, immunohistochemistry analyses were performed to evaluate the anti-metastasis mechanism of BA. As shown in Figure 3E and 3F, BA significantly inhibited the proliferation of nuclear Ki 67 positive cells. Besides, treatment of BA treatment of mice with BA inhibited the expression of MMP-2, MMP-9 and P-Stat3 in 4T1 tumor tissues. Overall, these data suggest that BA could inhibit breast cancer tissues by inhibiting cell proliferation and blocking metastasis,, which is consistent with the results *in vitro*.

### The tumor and lung metastatic environment were inhibited by BA

**Figure 4:**
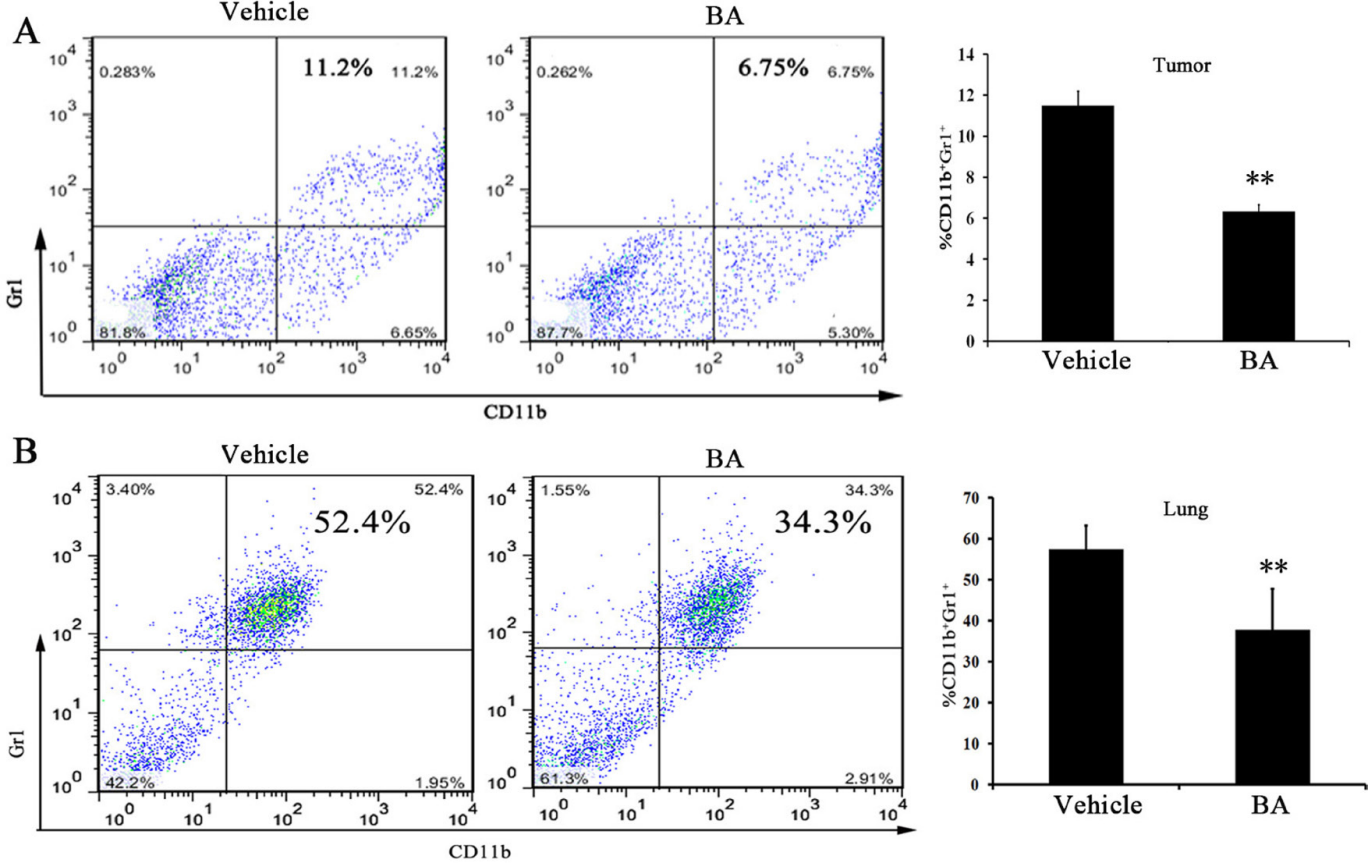
BA reduced lung and tumor Gr1^+^ CD11b^+^ MDSCs infiltration Gr1 ^+^ CD11b^+^ cells were gated and analyzed by FCM for the expression of MDSCs. (**A**) MDSCs isolated from the tumors of 4T1 tumor-bearing mice in day 21 were treated with vehicle or treated with BA at 10 mg/kg. (**B**) MDSCs isolated from the lungs of 4T1 tumor-bearing mice in day 21 were treated with vehicle or treated with BA at 10 mg/kg. Values represented mean ± SD (*n =* 6; ^**^P < 0.01)

MDSCs, as main feature by CD11b^+^ and Gr1^+^ double-positive myeloid cells in mice have been observed. Moreover, the accumulation of MDSCs into the tumors and lungs have an important role in the development of metastasis [30]. Meanwhile, MDSCs are closely related to a lung metastatic in patients with breast cancer [31–33]. Therefore, MDSCs in tumors and lung were quantified by FCM analyses in 4T1 tumor-bearing mice after 21 days of treatment. As shown in Figure 4A, the data showed that the percentage of MDSCs in tumor decreased in the 10 mg/kg-treated group compared with the control group. Moreover, we found that ∼20% reduction of MDSCs in the lung (Figure 4B) after 10 mg/kg of BA treatment. These results suggested that BA potently reduced the infiltration of MDSCs into the tumors and lung, which could inhibit tumor cell distant colonization.

### Safety profile of BA

**Figure 5:**
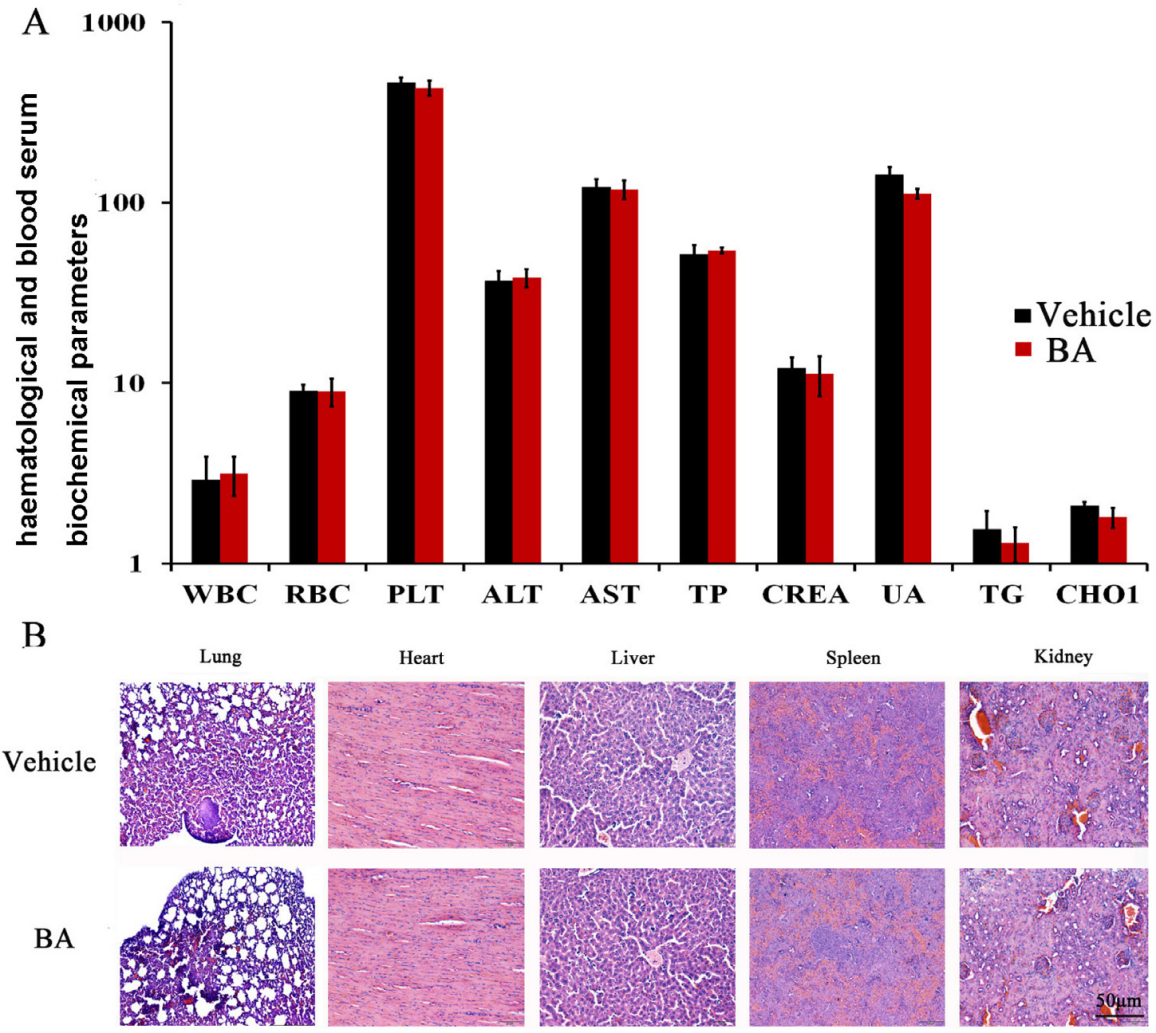
Evaluation of side effects of BA in mice (**A**) Hematological and serum biochemistry analysis of blood were done. Units of the parameters are as follows. WBC (white blood cell) and PLT (platelet), 10^9^/l; RBC (red blood cell), 10^12^/l; TP (total protein), g/l; ALT (alanine transarninase) and AST (aspartate aminotransferase), U/l ; CREA (creatinine) and UA (uric acid), μM; TG (triglyceride) and CHOL (cholesterol), mM. (**B**) BA did not cause obvious pathologic abnormalities in normal tissues. H&E staining of paraffin-embedded sections of the lung, heart, liver, spleen and kidney (20×).

As mentioned above, in the BA-treated groups, we did not observed skin ulceration, toxic death and body weight loss (Supplementary Figure 4D) in 4T1 tumor-bearing mice. To further elucidate the safety profile of BA, we determined whether the BA could cause the blood system’s abnormality. We performed blood chemistry analysis assay and blood routine analysis. Hematological and serum biochemistry analysis of the mice did not show any pathological changes by BA treatment (Figure 5A). Moreover, microscopic examination of the heart, liver, spleen and kidney showed no pathologic changes after treatment with BA (Figure 5B). Furthermore, histological analyses showed that the number of micrometastatic nodules in the BA-treated mice was decreased compared with that of untreated groups.

### BA-treated animals have fewer lung metastases

**Figure 6:**
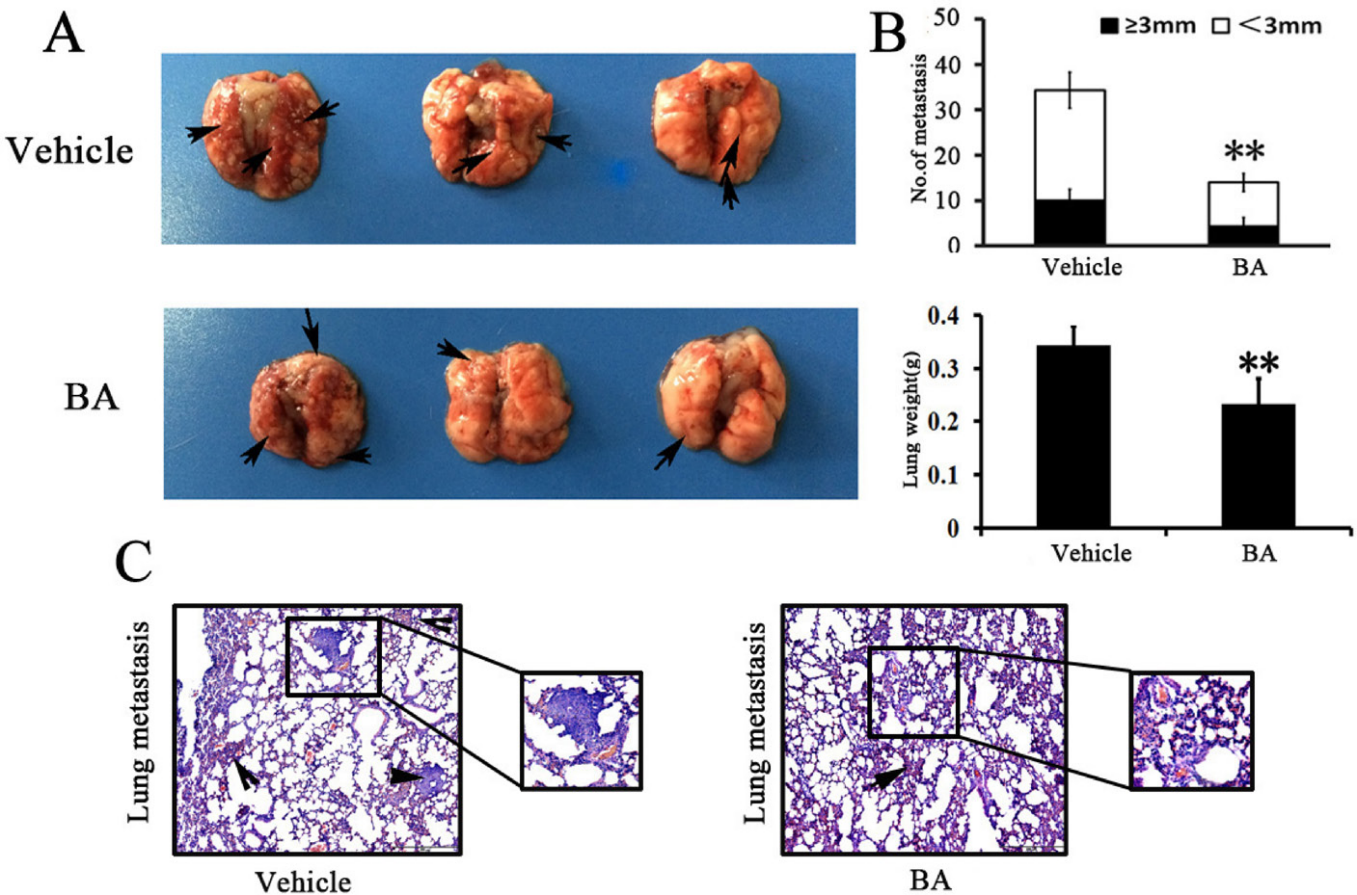
BA inhibited tumor metastasis (**A**) Lung metastatic nodules were visualized to show the inhibitory effects of BA on 4T1 intravenous inoculation model in 11 days after treatment. (**B**) The mean lung metastasis nodules and weight of lungs of each group, the treatment with BA at 10 mg/kg resulted in significant inhibition of lung metastasis versus vehicle control. Bars showed mean ± SD (*n =* 6; ^**^P < 0.01, ^***^P < 0.001). (**C**) Metastases count at day of sacrifice in lung of vehicle and BA-treated groups using H&E staining. Black arrowheads represented lung metastases.

Previous studies have showed that 4T1 breast tumor have a high metastatic potential and spontaneously metastasize to lung as early as 2 weeks after inoculation [34–36]. To analyze the effects of BA directly in the lungs, 4T1 cells were injected through the tail vein and, 5 days later, mice were treated with BA for 7 days. The lungs were removed and metastatic nodules were quantified. In vehicle-treated groups, multiple large metastasis nodules were evident, whereas the extent of lung metastasis nodules was markedly reduced in BA mice 10 mg/kg groups (Figure 6A and 6C). Moreover, there was a remarkable decrease in lung weight and numbers of nodules after BA treatment compared with the untreated control (Figure 6B). Overall, these results further indicated that BA could also inhibit the ability of 4T1 cells to grow in the lungs following tail vein injection.

## DISCUSSION

Breast cancer is highly malignant with considerable metastatic potential. Moreover, tumor metastasis possesses a predominant threat to cancer-related mortality and proliferation, migration and invasion of tumor cell are crucial for initial cancer metastasis. [27, 28] Therefore, it is still needed to discover the novel potential drug candidate to prevent tumor metastasis. In this study, we evaluated whether BA could inhibit the tumor cell migration and invasion for blocking tumor metastasis. Our results showed that BA could inhibit breast cancer cell vitality with low micromole by MTT and clonogenicity assays. From the wound healing migration and invasion assays, our data showed that BA inhibited MDA-MB-231 and 4T1 cell migration and invasion *in vitro*.

Since the outstanding inhibition effect of BA in proliferation, migration and invasion, we examined the anti-metastasis effects of BA in our established 4T1 tumor model and 4T1 caudal vein model in BALB/c mice. To our delight is that administration of BA at the dose of 10mg/kg significantly inhibited breast cancer metastasis to lungs. The results also showed that tumor growth was inhibited by BA administration (10 mg/kg/day). It has been reported that focal adhesion kinase(FAK)/MMP-involved pathway is critical for cancer invasion and metastasis [27]. MMP-2 and MMP-9 activation can particularly enhance tumor cell metastatic potential in breast cancer [37]. On the other hand, MMPs have a positive relationship to invasive behavior of tumors, and tissue inhibitor of MMP-2(TIPM2) which could inhibit activity of MMPs is considered to have inhibitory effect on tumor metastasis [27, 38, 39]. In our study, BA inhibited MMP-2 and MMP-9 expression and simultaneously activated TIMP2 in 4T1 and MDA-MB-231 cells. Meanwhile, IHC results showed that BA inhibited the expression of MMP-2 and MMP-9 *in vivo*. The results suggested that BA could inhibit breast cancer cell invasion and metastasis by blocking MMPs.

Recent studies have also reported that activated Stat3 were closely associated with the development of breast cancer. In addition, constitutive Stat3 also has an important role in controlling cell migration and invasion, suggesting that targeting Stat3 might be a potentially important new form of breast cancer therapy [40]. In the present study, we found that BA not only decreased expression of Stat3 phosphorylation at tyrsione residue 705 but also downregulated P-FAK, MMP-2 and MMP-9 expression in 4T1 and MDA-MB-231 cells. Meanwhile, IHC assay results also showed that BA inhibited the expression of P-Stat3 *in vivo*. These results suggest that BA has significant antitumor and antimetastasis effects, with at least inhibition of Stat3 signaling pathway.

A lot of evidences suggest that the tumor microenvironment can promote tumor development, progression and immune evasion [41, 42]. In particular, MDSCs are important components of the tumor microenvironment. Moreover, MDSCs are present in lots of patients and experimental animals with cancer that decrease immune surveillance and antitumor immunity [43]. In this study, CD11b^+^/Gr-1^+^ MDSCs in tumors and lungs were quantified by FCM analyses in tumor-bearing mice after BA treatment. Our results indicated that compared with vehicle-treated group, the treatment of mice with BA caused a remarkable decrease in the number of MDSCs in the tumors and lungs. Therefore, it is conceivable that BA can not only potentiate the antitumor effects but also suppress the lung metastasis.Furthermore, our results implied that BA could inhibit lung metastasis in the 4T1 intravenous inoculation model. These results were consistent with the findings *in vitro* and *in vivo*, suggesting that breast tumor metastasis inhibited by BA could be mainly ascribed to the impediments of cancer cell migration and invasion.

To sum up, our current project was conducted to investigate the effects of BA on breast cancer metastasis and elucidate its underlying mechanism. As far as we know, this is the first study to demonstrate that BA could block breast cancer cells migration and invasion. Importantly, BA could inhibit lung metastasis by reducing the number of MDSCs in the tumors and lungs. Therefore, these results implied that BA might be a potential therapeutic agent for blocking breast cancer growth and metastasis.

## MATERIALS AND METHODS

### Reagents and antibodies

BA was purchased from (Mansite Chemical Co., Cheng Du, Si chuan, China.). Purity (98.9%) was measured by high-performance liquid chromatograph(HPLC) analysis. For *in vitro* assays, BA was prepared initially as a 20mM stock solution in dimethyl sulfoxide (DMSO) and stored at –20° C. Then the stock solution diluted in the relevant assay medium, and 0.1% DMSO served as a vehicle control. For *in vivo* studies, BA was prepared in 5% DMSO in corn oil, and 5% DMSO in corn oil served as a vehicle control.

MTT and DMSO were bought from Sigma Chemical Co. (St Louis, MO, USA). The primary antibodies against MMP-2, MMP-9, Tissue inhibitor of MMP-2(TIMP-2), cleaved caspase-3 (CC-3), FAK, P-FAK, Stat3, P-Stat3 and β-actin were purchased from Cell Signaling Technology (Beverly, MA, USA). FITC-CD11b, PE-Gr1 conjugated antibodies were obtained from BD Biosciences (San Diego, CA, USA).

### Cell culture

The human breast cancer cell lines, MCF-7 and MDA-MB-231, the mouse mammary carcinoma cell line 4T1, were obtained from the American Type Culture Collection (Rockville, MD, USA). Cells were propagated in RPMI 1640 or DMEM media containing 10% heat-inactivated fetal bovine serum (FBS; Hyclone, Logan, UT, USA) and 1% antibiotics (penicillin and streptomycin) in 5% CO_2_ at 37° C.

### Cell viability assay

The cell viability of BA-treated breast cancer cells was assessed by MTT assay. In brief, the exponentially growing cells (2∼ 5 × 10^3^/well) were plated in 100 μl/well in 96-well plates. After 24 h incubation, the cells were treated with different concentrations of BA. After treatment for 48 and 72 h, respectively, 20 μl of 5 mg/ml MTT was added to each well, and the plates were incubated at 37° C for additional 4 h. The medium was subsequently removed, the purple-colored precipitates of formazan were dissolved in 150 μl of DMSO. The color absorbance was recorded at 570 or 492 nm using a Spectra MAX M5 microplate spectrophotometer (Molecular Devices, CA, USA). All experiments were performed in triplicate.

### Colony formation assay

Colony formation assay was measured as previously described [40]. In brief, 4T1, MCF-7 and MDA-MB-231 cells were seeded in specified numbers (300–500 cells/well) in six-well plates. After 24 h incubation, the cells were treated with various concentrations of BA and then incubated for additional 12 days. Then the cells were fixed with methanol and stained with a 0.01% crystal violet solution for 30 min, and the colonies (600 cells) were counted under microscope. Data shown represent the average of three independent experiments.

### Western blot analysis

The western blot analysis was performed as described previously, with minor modification [30]. In brief, 4T1 and MDA-MB-231 cells were treated with BA in designed concentration for 48 h, then, cells were washed twice with cold PBS and lysed in RIPA buffer. The protein concentrations were measured using the Lowry method and equalized before loading. Equal amounts of protein from each sample were subjected to sodium dodecyl sulfate-polyacrylamide gel electrophoresi gels and transferred onto polyvinylidene difluoridemembranes (Amersham Bioscience, Piscataway, NJ, USA). Then, the membranes were blocked for 2 h at 37° C and incubated with specific primary antibodies overnight at 4° C. After incubation with the relevant secondary antibodies, the reactive bands were identified using an enhanced chemiluminescence kit (Amersham).

### Wound-healing migration assay

Wound-healing migration assay was performed as described previously [27, 30]. When cancer cells grew to 90% confluence, cell monolayer was scraped by sterile 0.1 ml pipette tips, and fresh medium which contains only 1% FBS was added containing different concentrations of BA [44]. After 48 h incubation, cells were fixed and photographed. Images were acquired using a microscope (Zeiss, Jena, Germany) and the percentage inhibition of migrated cells was expressed using 100% as the value assigned for untreated group.

### Boyden chamber migration and invasion assay

Boyden chamber (8 μm pore size) migration assay was performed as previously described, with some modification [30, 45]. In brief, a total of 1 × 10^5^ cells (for 4T1) or 5 × 10^4^ cells (for MDA-MB-231) in 100μl serum-free medium were added in the upper chamber, and 600μl of medium containing 10% FBS was added at the bottom. Different concentrations of BA were added in both chambers. Cells were allowed to migrate for 24 and 48 h. Non-migrated cells in the upper chamber were discarded using a cotton swab. The migrated cells were fixed in methanol and stained with 0.5% crystal violet. Migrated cells in six randomly selected fields were counted and photographed under a light microscope. Invasion assay was performed according to previous studies [30, 45]. In brief, the upper surface of the transwell membrane were coated with serum-free medium diluted Matrigel (1:3, 60 μl/well, BD Biosciences) and the lower compartment of the chambers were filled with 600 μl medium with 10 % FBS. 2 × 10^5^ cells (for 4T1) or 8 × 10^4^ cells (for MDA-MB-231) in 100 μl serum-free medium were placed in the upper part of each transwell and treated with different concentrations of BA. After incubation for 48 h, cells on the upper side of the filter were removed. Cells located on the underside of the filter were fixed with methanol and stained with 0.5% crystal violet, then, migrated cells were counted and photographed under a light microscope. The results were expressed as the percentage inhibition rate of migration compared with untreated group.

### Flow cytometry

Single-cell suspensions from lungs and tumors were prepared as described previously [46]. Then 1 × 10^6^ freshly prepared cells were stained with fluorochrome-conjugated CD11b and Gr1 antibodies. Data were collected by FCM and analyzed with Flow Jo software.

### Mice and tumor model

Female BALB/c mice (6–8 weeks old) were obtained from HFK bioscience CO., LTD, Beijing, China. All animal experiments were approved by the Ethics Committee and Institutional Animal Care and Treatment Committee of Sichuan University (Chengdu, China). In order to verify our results, we used two animal models. One is the subcutaneous immunization model [30]. We inoculated female mice with 4T1 cell (1.0 × 10^6^/100 μl/each).When visible tumors (∼80 mm^3^) had been developed at the injection sites, the tumor-bearing mice were randomized into two groups (six mice per group), and received intraperitoneally injection of BA 10 mg/kg or vehicle, respectively, once daily for 21 days. Tumor volumes and body weight were measured every 3 days. The tumor size was calculated according to the formula: Tumor volume (mm^3^) = 0.5 × L × W^2^ where L is the length and W is the width. At the termination of the experiment, all animals were euthanized by cervical dislocation. At that time tumors and internal organs, such as the heart, livers, spleens, lungs and kidney were excised from animals. Another is intravenous inoculation model. We inoculated female mice with 4T1 cell (2.0 × 10^5^ cells/100 μl/mouse). After ∼5 days, the tumor-bearing mice were randomized into two groups (six mice per group), and received intraperitoneally injection of BA 10 mg/kg or vehicle, respectively, once daily for 10 days. At the end point, mice were sacrificed and lungs were dissected.

### Immunohistochemistry

IHC staining of tumor sections were described previously [30]. One part of paraffin tumor sections and lungs sections were stained with hematoxylin and eosin (H&E). The other part paraffin tumor sections were stained with CC-3, Ki-67, MMP-2 and MMP-9 antibodies. Images were taken with Leica microscope (Leica, DM4000B).

### Toxicity evaluation

To investigate potential side effects or toxicity on mice during the treatment, all the animals were observed continuously for relevant indexes such as body weight, diarrhea, anorexia and other clinical symptoms. On the 21st day, all animals were euthanized by cervical dislocation after taking blood from eyeball. Blood was obtained for blood routine analysis and blood chemistry analysis. The tissues of heart, liver, spleen, lung and kidney were stained with H&E for histopathologic examination.

### Statistical analysis

All experiments were performed at least in triplicate. Data were represented as mean ± SD of three independent experiments. *P*-values for comparison of two groups were determined by two-tailed Student’s *t*-test. Statistically significant *P*-values were labeled as follows: ^*^P < 0.05; ^**^P < 0.01; ^***^P < 0.001.

## SUPPLEMENTARY MATERIALS FIGURES


