# Supplementary material for: Betulinic acid impairs metastasis and reduces immunosuppressive cells in breast cancer models
Source: Oncotarget. 2017 Dec 17;9(3):3794–804. doi: 10.18632/oncotarget.23376 (PMC5790500; doi:10.18632/oncotarget.23376)
Supplement: Supplementary file 1 [file oncotarget-09-3794-s001.pdf]

Betulinic acid impairs metastasis and reduces immunosuppressive cells in breast cancer models

SUPPLEMENTARY MATERIALS

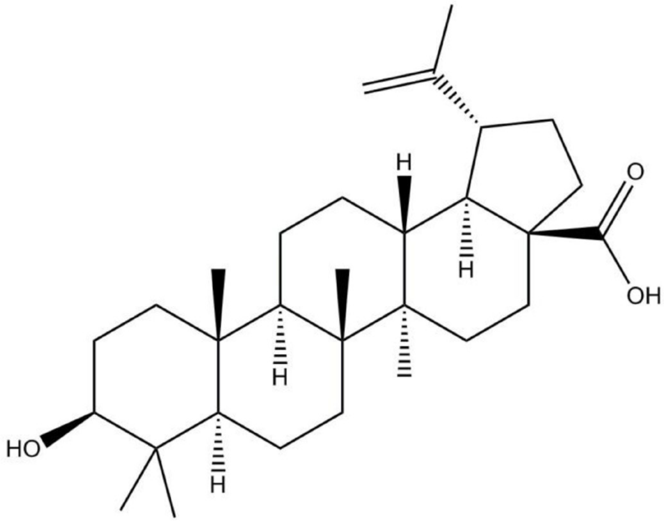

Supplementary Figure 1: The chemical structure of betulinic acid (BA).

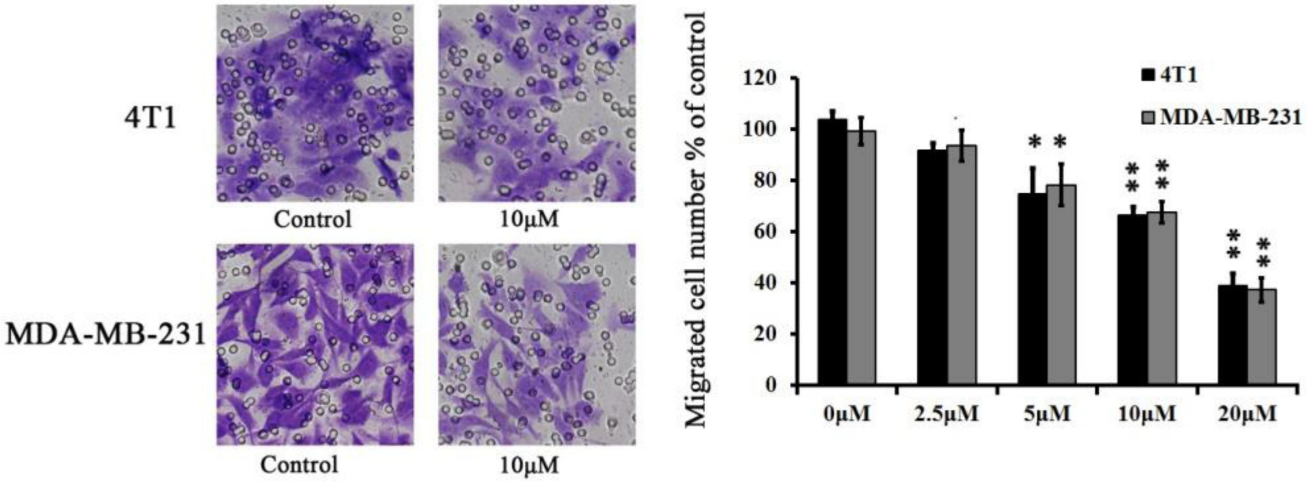

Supplementary Figure 2: BA inhibits breast cancer cells 4T1 and MDA-MB-231 migration and invasion in 24 h.

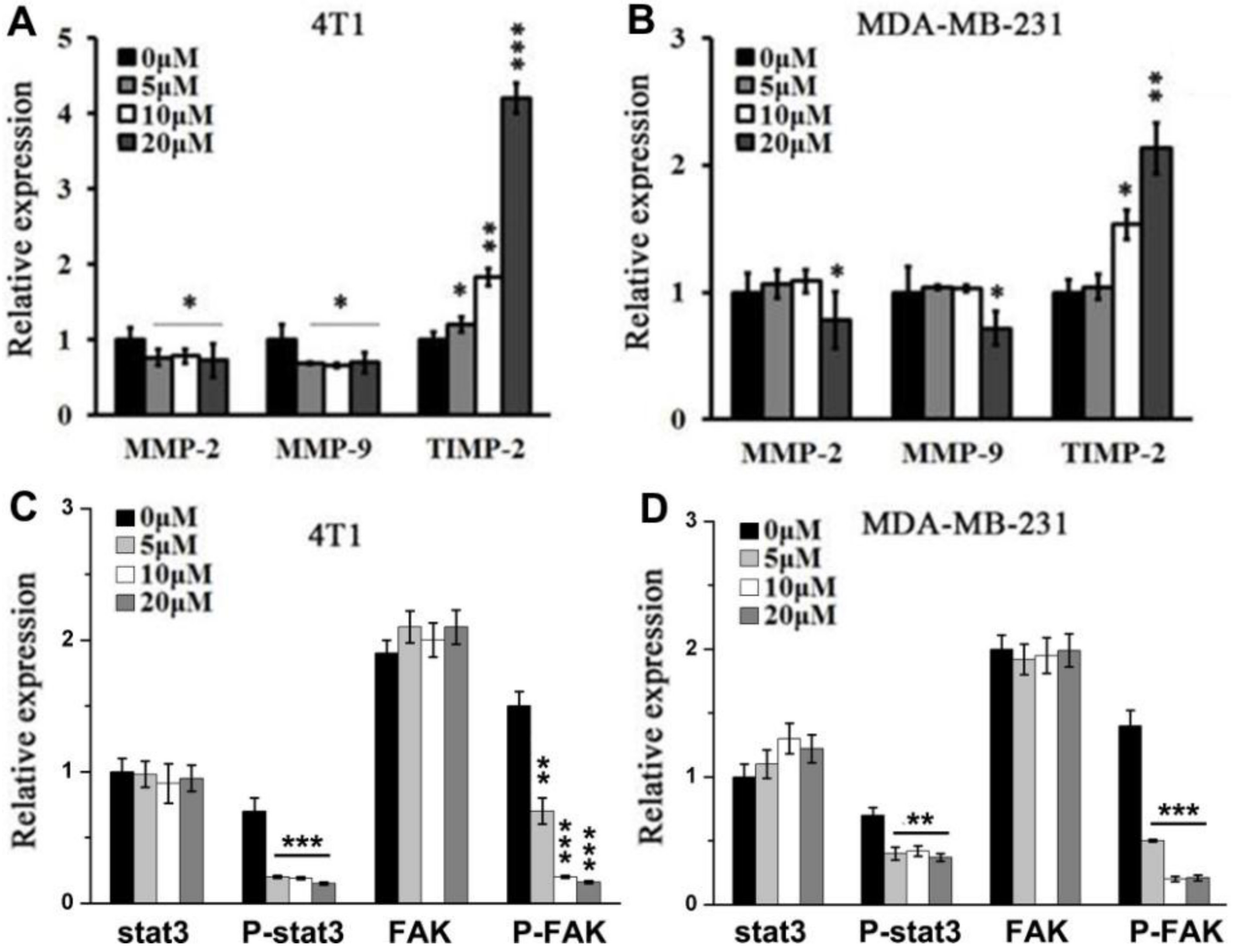

Supplementary Figure 3: The relative expression of MMP-2, MMP-9,TIMP-2, Stat3, P-Stat3, FAK and P-FAK of 4T1 and MDA-MB-231.

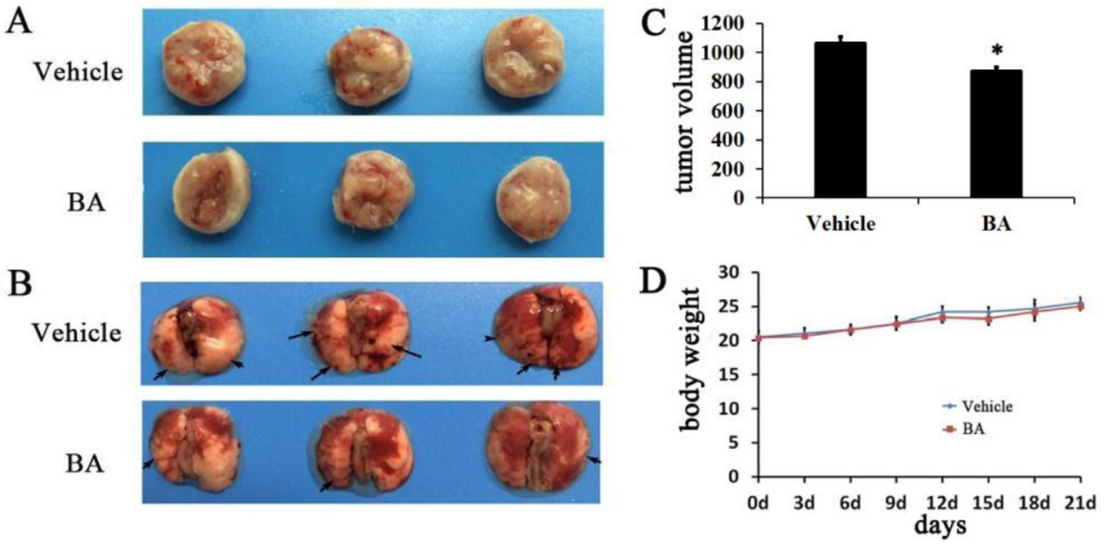

Supplementary Figure 4: Anti-tumor and lung metastasis effects of BA *in vivo* in subcutaneous tumor model. (**A**) The tumor size of vehicle and BA-treated groups. (**B**) The lungs from the mice at day 21 in vehicle and BA-treated groups. (**C**) The tumor volume of vehicle and BA-treated groups. (**D**) The body weight were measured and calculated every three days.
